# Hair, Wool, Rags, and Thread Expelled in a Mass from the Rectum

**Published:** 1847-11

**Authors:** 


*Hair, Wool, Rags, and Thread expelled in a Mass from the Rectum.*—Mr. Edward Spry, of the Royal Cornwall Infirmary, recently read, at the annual meeting of the South Western Branch of the Provincial Association, an account of the cure of a young woman, aged sixteen, who voided with excessive pain two large lumps of foreign matters—the first about the size of an ordinary pullet's egg, the second larger and longer—much compressed and very hard, having a thin albuminous coating which served to conceal the character of the contents of the lumps until forcibly separated. After being washed they were found to consist of dyed wool of various colours; of thread, of worsted, and of cotton and linen rags, all compactly fitted together, and weighing one ounce and four drachms. The patient had never from infancy enjoyed a good state of health, complaining of severe pain in her bowels. In September, 1846, when first attended by Mr. Spry, there was a considerable swelling just below the margin of the right ribs, and occupying the whole of the right hypochondrium. This tumour had a well-defined edge extending towards the epigastrium, and firm pressure on it occasioned pain. There were occasional vomitings. After the evacuation of the foreign substances, she steadily improved in health, and appears a fresh-coloured girl. It appears that this patient has no recollection of swallowing any part of the matters evacuated, but her mother stated that when a child learning to walk she would get at the seat of a settle in the kitchen, by the edge of which she held, and that the children often amused themselves by turning over the contents of a box in which old rags of all sorts were deposited; and that then the child must have swallowed the substances which after the lapse of many years were voided by the rectum.—*Ibid.*

